# A Case of Suspected Adverse Reactions to Sirolimus in the Treatment of Generalized Lymphatic Anomaly

**DOI:** 10.1155/2019/3101357

**Published:** 2019-04-30

**Authors:** Takayuki Fujii, Ryuichi Shimono, Aya Tanaka, Hiroto Katami

**Affiliations:** Department of Pediatric Surgery, Faculty of Medicine, Kagawa University, 1750-1 Ikenobe, Mikicho, Kitagun, Kagawa 761-0793, Japan

## Abstract

Generalized lymphatic anomaly (GLA) is characterized by diffuse or multicentric proliferation of dilated lymphatic vessels resembling common lymphatic malformation, and thoracic lesions can be related to a poor prognosis. Sirolimus, an inhibitor of the mammalian target of rapamycin, is effective against vascular anomalies with few severe adverse drug reactions. Here, we report the case of a patient with intractable hemothorax pleural effusion due to GLA who was treated with sirolimus and experienced disseminated intravascular coagulation. Although a standard treatment for GLA has not been established, pleural fluid might be reduced using the Kampo medicine Eppikajyutsuto.

## 1. Introduction

Generalized lymphatic anomaly (GLA) is characterized by diffuse or multicentric proliferation of dilated lymphatic vessels resembling common lymphatic malformation, and thoracic lesions can be related to a poor prognosis [1].

In recent years, several studies have reported that sirolimus, an inhibitor of the mammalian target of rapamycin (mTOR), is effective and well tolerated in the treatment of vascular anomalies [2–4]. Adverse drug reactions to sirolimus include leukopenia, mucositis, gastrointestinal manifestations, and hyperlipidemia; however, severe adverse drug reactions are rare [2–4].

Here, we report the case of a patient with intractable hemothorax pleural effusion due to GLA who was treated with sirolimus and developed disseminated intravascular coagulation (DIC). Although a standard treatment for GLA has not been established, pleural fluid might be reduced using a Kampo medicine called Eppikajyutsuto (TJ-28; Tsumura & Co., Tokyo, Japan), which is reportedly effective against lymphatic malformations (LMs) [5–7].

## 2. Case Presentation

**Figure 1:**
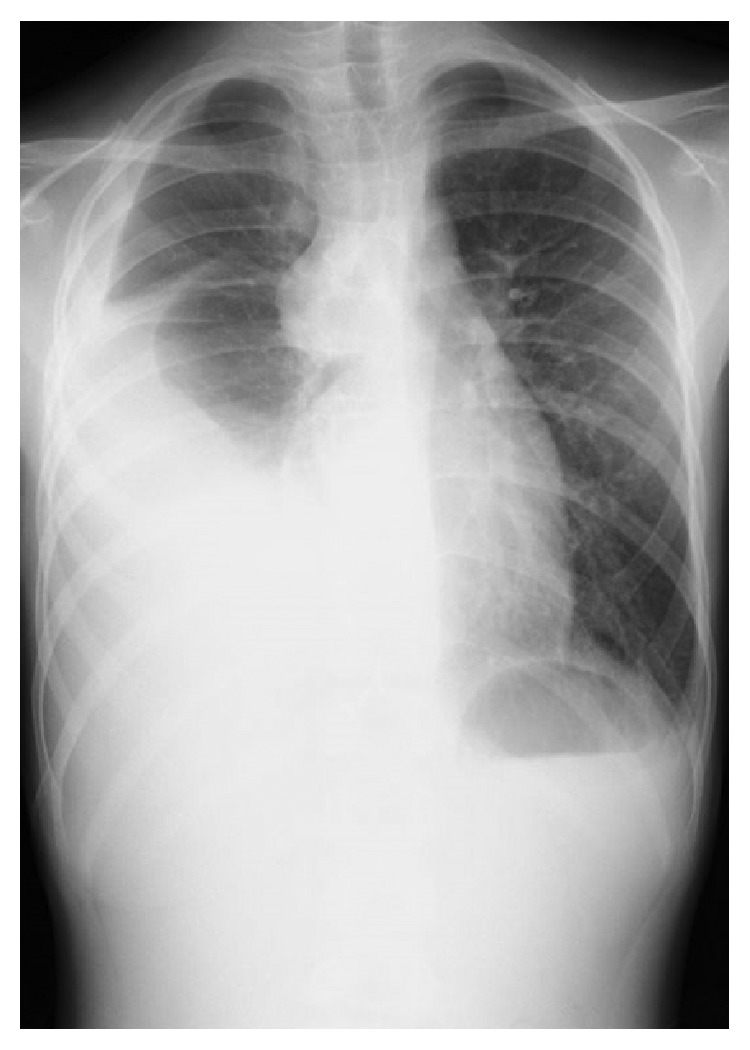
A radiograph showing massive pleural effusion in the right lung.

**Figure 2:**
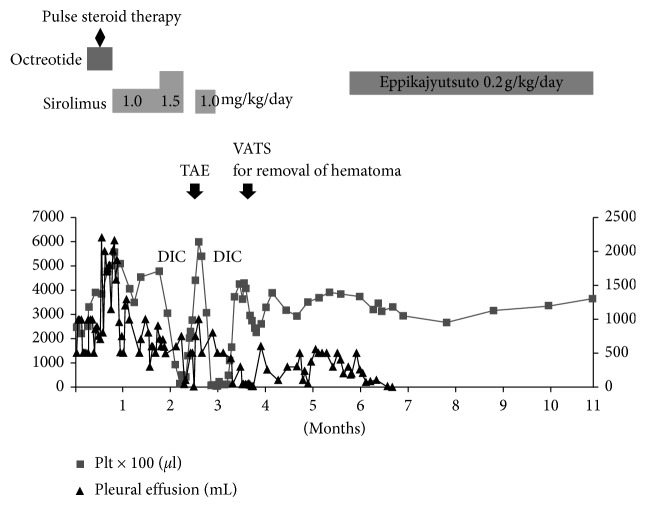
Clinical course of treatment in a 13-year-old male patient. (■) platelets (Plt); (▲) pleural effusion. TAE, transcatheter arterial embolization; VATS, video-assisted thoracic surgery; DIC, disseminated intravascular coagulation.

**Figure 3:**
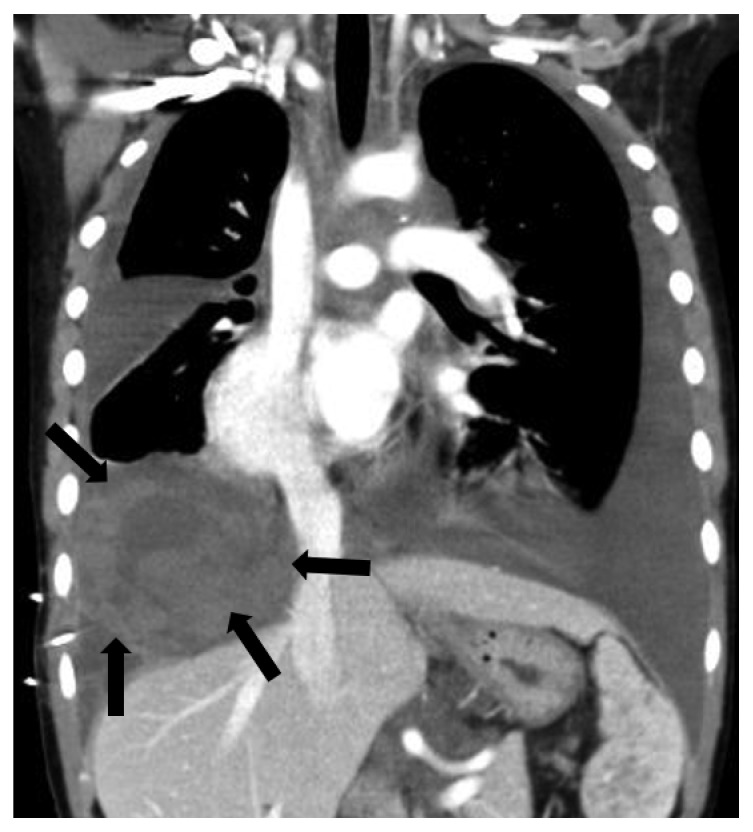
A chest contrast-enhanced computed tomography scan showing massive pleural effusion in both lungs. The black arrow shows a high-density hematoma in the right lung.

A 13-year-old boy underwent pericardial fenestration and thoracic duct ligation for pericardial and pleural effusion at 3 years of age and was diagnosed with GLA after a pleural biopsy. The patient experienced no pleural effusion before his 11^th^ birthday. The patient had a history of cerebrospinal fluid leakage due to a skull fracture at 7 years of age. The patient was referred to our department immediately following pleural effusion when he was 11 years old. A hematological examination showed high values for D-dimer (22.2 *μ*g/mL) and P-FDP (50.9 *μ*g/mL). A radiograph showed pleural effusion in the right lung (Figure 1). Thoracentesis revealed chylothorax mixed with blood components. Magnetic resonance imaging showed additional lesions on the lymph ducts on both sides of the inner auditory channels; computed tomography (CT) showed diffuse osteolytic changes on both sides of the femoral neck and thoracic vertebra.  Figure 2 shows the patient's clinical course. Although the patient abstained from eating and parenteral nutrition was provided in addition to octreotide testing and pulse steroid therapy, pleural effusion worsened and became bilateral. Two or more liters were drained on days when there was a large amount of pleural effusion. We were unable to locate the site of the leakage even though we conducted a lymphogram to treat the pleural effusion and identify the leakage site. Sirolimus administration was initiated at 0.88 mg/m^2^/day, which proved to be an insufficient dosage. However, when the dosage was increased to 1.3 mg/m^2^/day after 1 month, the patient experienced an onset of disseminated intravascular coagulation (DIC) after 1 week. At that time, a blood examination showed platelet (1.4 × 10^4^/*μ*L), P-FDP (590 *μ*g/mL), fibrinogen (114 mg/dL), prothrombin time rate (1.35), antithrombin (129%), and no liver dysfunction. The urine and blood cultures were negative. Viral serology was negative for cytomegalovirus, and aspergillus antigen was negative. Rheumatoid factor and antinuclear antibody were normal levels. The CT scan showed no sign of pneumonia or pyothorax. We diagnosed him with DIC using DIC score [8]. Although we temporarily paused the administration of sirolimus, the patient experienced an additional onset of DIC 10 days after we resumed administration. Thus, he underwent thoracoscopic removal of the hematoma (Figure 3). The trough level of sirolimus during administration was 3.4–8.9 ng/mL, which does not represent abnormal elevation. Although an additional pause in the administration of sirolimus did not reduce the amount of pleural fluid, there were no additional onsets of DIC. Subsequently, there was a significant decrease in pleural fluid once Eppikajyutsuto was administered at 0.2 g/kg/day. We were able to proceed with tube thoracostomy removal 40 days after the initiation of oral administration. There was no re-accumulation of pleural fluids in the 18 months following the initiation of oral administration.

## 3. Discussion

In total, 89.2% of GLA cases involve chest lesions. Many cases are intractable despite drainage of the thoracic cavity, pleurodesis, and steroid treatment [1].

In recent years, sirolimus has been considered as a new therapeutic option for vascular anomalies [2–4]. mTOR is a serine threonine kinase regulated by phosphoinositide 3 kinase (PI3K) and protein kinase B (Akt). The PI3K/Akt/mTOR pathway is the basis for cell growth and proliferation and also increases the expression of vascular endothelial growth factor, regulating angiogenesis as well and lymphangiogenesis. mTOR inhibitors directly inhibit mTOR, blocking downstream protein synthesis and eliciting antitumoral and antiangiogenic effects [2, 9].

Adverse drug reactions to sirolimus include leukopenia, mucositis, gastrointestinal manifestations, and hyperlipidemia; however, severe adverse drug reactions that threaten life are rare [2–4]. The range of sirolimus concentration appropriate for the treatment of vascular anomalies was reported as 5–15 ng/mL [2]. Our patient had no abnormal high blood levels of sirolimus. GLA including kaposiform lymphangiomatosis may cause the severe thrombocytopenia and coagulopathy. Therefore, it might be associated with the patient's diseases. However, our patient experienced DIC consistent with the timing of sirolimus administration. Although DIC has not been reported as a side effect of sirolimus, we believe that it is important to note the onset of DIC when using sirolimus. On the other hand, Hammer et al. reported that sirolimus decreased coagulation abnormalities [4]. Further studies are needed to elucidate the mechanism of the effect of sirolimus on the blood coagulation system.

The Kampo medicine Eppikajyutsuto was created to conform to other Kampo formulations such as Mao, Sekko, and so on. It has an irrigation effect that normalizes the disequilibrium of fluids in the body. It is also reported to have an anti-inflammatory effect and is effective against lymphatic malformations [5–7]. Mao (ephedra herb) is a main component of Eppikajyutsuto. The herb ephedra is suggested to suppress of vascular endothelial growth factor activity by inhibiting prostaglandin E2 synthesis and cyclooxygenase protein synthesis, which might contribute to the shrinkage of LMs [7, 10–12]. Hashizume et al. reported the mean LM volume shrinkage rate of 54.5 ± 38.3% in eight patients who received the medicine [6].

In the present case, because we performed various treatments, it was difficult to determine which treatment was effective in reducing pleural effusion. However, because pleural effusion decisively decreased after the administration of Eppikajyutsuto and we were able to proceed with tube thoracostomy removal, we assume that Eppikajyutsuto influenced the disequilibrium of fluids in the body (pleural effusion) in this patient. Further research based on this finding is necessary.
